# PGC-1α modulates denervation-induced mitophagy in skeletal muscle

**DOI:** 10.1186/s13395-015-0033-y

**Published:** 2015-03-18

**Authors:** Anna Vainshtein, Eric MA Desjardins, Andrea Armani, Marco Sandri, David A Hood

**Affiliations:** Muscle Health Research Centre, School of Kinesiology and Health Science, York University, 4700 Keele St., Toronto, Ontario M3J 1P3 Canada; Department of Biomedical Sciences, University of Padova, Viale G. Colombo 3, I-35121 Padova, Italy; Venetian Institute of Molecular Medicine, 35129 Padova, Italy; Telethon Institute of Genetics and Medicine (TIGEM), 80131 Napoli, Italy

**Keywords:** Autophagy, Mitophagy, Muscle atrophy, PGC-1α, Disuse, Mitochondria, Mitochondrial turnover, TFEB

## Abstract

**Background:**

Alterations in skeletal muscle contractile activity necessitate an efficient remodeling mechanism. In particular, mitochondrial turnover is essential for tissue homeostasis during muscle adaptations to chronic use and disuse. While mitochondrial biogenesis appears to be largely governed by the transcriptional co-activator peroxisome proliferator co-activator 1 alpha (PGC-1α), selective mitochondrial autophagy (mitophagy) is thought to mediate organelle degradation. However, whether PGC-1α plays a direct role in autophagy is currently unclear.

**Methods:**

To investigate the role of the co-activator in autophagy and mitophagy during skeletal muscle remodeling, PGC-1α knockout (KO) and overexpressing (Tg) animals were unilaterally denervated, a common model of chronic muscle disuse.

**Results:**

Animals lacking PGC-1α exhibited diminished mitochondrial density alongside myopathic characteristics reminiscent of autophagy-deficient muscle. Denervation promoted an induction in autophagy and lysosomal protein expression in wild-type (WT) animals, which was partially attenuated in KO animals, resulting in reduced autophagy and mitophagy flux. PGC-1α overexpression led to an increase in lysosomal capacity as well as indicators of autophagy flux but exhibited reduced localization of LC3II and p62 to mitochondria, compared to WT animals. A correlation was observed between the levels of the autophagy-lysosome master regulator transcription factor EB (TFEB) and PGC-1α in muscle, supporting their coordinated regulation.

**Conclusions:**

Our investigation has uncovered a regulatory role for PGC-1α in mitochondrial turnover, not only through biogenesis but also via degradation using the autophagy-lysosome machinery. This implies a PGC-1α-mediated cross-talk between these two opposing processes, working to ensure mitochondrial homeostasis during muscle adaptation to chronic disuse.

**Electronic supplementary material:**

The online version of this article (doi:10.1186/s13395-015-0033-y) contains supplementary material, which is available to authorized users.

## Background

Skeletal muscle is the largest organ of the body and as such is recognized for essential roles that extend beyond locomotion. Muscle is an indispensable metabolic center that possesses a remarkable capacity to adapt to alterations in its milieu, a property known as muscle plasticity. This type of malleability to cues such as contraction, nutrient availability, or hormonal stimuli requires efficient cellular remodeling and a rapid shift in metabolic profile. Since mitochondria are central to muscle metabolism, these types of alterations require amendments in organelle content and its network. Mitochondrial density depends on the intricate balance between biogenesis and degradation. Biogenesis is largely regulated transcriptionally through the coordinate expression of nuclear and mitochondrial genes, governed by the transcriptional co-activator peroxisome proliferator gamma coactivator-1α (PGC-1α) [1]. On the other hand, mitochondrial degradation is achieved through a selective form of macroautophagy (hereafter autophagy) termed mitophagy [2]. This process is of particular importance for long-lived post-mitotic tissues such as the striated muscle and neurons, as this represents the sole mechanism for these cells to rid themselves of dysfunctional organelles. During mitophagy, the defective mitochondria are first segregated from the network and are then engulfed into double-membrane vesicles termed autophagosomes [3], which are subsequently delivered to the lysosome for proteolytic degradation.

Mitochondrial health is vital not only for proficient energy provision but also for proper cellular signaling and homeostasis, as mitochondria are often found at the fulcrum of cellular life-and-death decisions [4]. Thus, it is not surprising that mitochondrial abnormalities have been implicated in a plethora of muscle wasting conditions such as the sarcopenia of aging [5], pathology-related cachexia, [6] and various muscular dystrophies [7-9]. Interestingly, the skeletal muscle from animals with autophagic deficiencies closely resembles that of sarcopenic and atrophic patients [10]. Thus, the intricacies underlying mitochondrial remodeling in the skeletal muscle have great therapeutic potential for a myriad of debilitating conditions. While mitophagy is important for proper tissue remodeling and organelle turnover, the regulation of this process in the skeletal muscle remains largely elusive.

PGC-1α, which is most well recognized for its role in mitochondrial biogenesis, has been documented to spare muscle mass and improve endurance in atrophic muscle induced by senescence [11], chronic heart failure [12], and a variety of additional muscle wasting conditions [13,14]. More recently, PGC-1α has been implicated in the autophagy-lysosome pathway, and its overexpression was demonstrated to induce lysosomal biogenesis, possibly through the upregulation of transcription factor EB (TFEB) [15-17], a transcription factor that is a master regulator of the lysosomal system. However, the role of PGC-1α in mitochondrial removal and autophagy in the skeletal muscle has not been thoroughly examined. To this end, the purpose of this study was to examine the possibility of a coordinated regulation of mitochondrial remodeling by the metabolic master regulator PGC-1α in the skeletal muscle. Here, we investigate the involvement of PGC-1α in basal and denervation-induced autophagy using both gain- and loss-of-function approaches. Our results implicate PGC-1α in the regulation of the mitochondrial network, not only via biogenesis but also through degradation.

## Methods

### Animal generation, procedures, and treatment

The generation and characterization of PGC-1α knockout (KO) and PGC-1α transgenic (Tg) mice have been described in detail elsewhere [13,18-20]. PGC-1α whole-body KO animals were generated by Lin *et al.* as described previously [18]. For PGC-1α Tg mice, the transgene was expressed specifically in the muscle under the control of muscle creatine kinase (MCK) promoter. All mice were housed in a 12:12-h light-to-dark cycle and given food and water *ad libitum*. Where indicated, the animals were unilaterally denervated by severing the sciatic nerve as previously described [21], while the contralateral limb served as an internal control. To assess autophagy flux, the animals were treated with either colchicine or an equal volume of vehicle (water) through an intraperitoneal injection every 24 h at a dose of 0.4 mg/kg/day [22] for the last 4 days of denervation, with the final injection taking place 24 h prior to sacrifice. Following 7 days of denervation, the muscles were harvested and either immediately frozen for histology, protein, and gene expression analysis or used for cellular fractionation. Extensor digitorum longus (EDL) muscles were fixed for single fiber analysis or electron microscopy. All procedures involving PGC-1α KO and corresponding wild-type (WT) animals were approved by and conducted in accordance with the regulations of the York University Animal Care Committee in compliance with the guidelines set forth by the Canadian Council on Animal Care. All PGC-1α Tg and corresponding WT procedures were approved and authorized by the Italian Ministry of Health.

### Histology and cross sectional area

Cytochrome oxidase (COX) and succinate dehydrogenase (SDH) staining was performed on 10-μm cross sections of digitorum longus (EDL) and tibialis anterior (TA) muscles as previously described [23]. Fiber cross-sectional area (CSA) of individual muscle fibers was determined using Image J software (NIH, Bethesda, MD, USA) by a blinded investigator. Fiber sizes were expressed in micrometers squared.

### COX activity

COX enzyme activity was measured as previously detailed [24] by determining the maximal rate of oxidation of fully reduced cytochrome *c*, evaluated as a change in absorbance at 550 nm using a microplate reader (Bio-Tek Synergy HT, BioTek Instruments, Inc., Winooski, VT, USA).

### Electron microscopy

Tissue preparation for electron microscopy (EM) was performed as previously described [25]. Briefly, sections of EDL muscles from WT and KO animals were fixed for 1 h in 3.0% glutaraldehyde followed by a 1-h fixation in 1% osmium tetroxide diluted in 0.1 M sodium cacodylate at room temperature. The muscle sections were dehydrated and embedded in Epon resin, sliced into ultrathin (60-nm) sections, and stained with uranyl acetate and lead citrate. Electron micrographs were obtained using a Philips EM201 electron microscope (Philips, Amsterdam, The Netherlands).

### Gene expression analysis

Quantitative real-time PCR was performed to determine mRNA expression levels. Total RNA was isolated using TRIzol reagent (Invitrogen, 15596-026, Life Technologies, Grand Island, NY, USA). RNA was reverse transcribed into cDNA using a Superscript III first strand synthesis kit (Invitrogen, 18080-044) according to manufacturer instructions. The primers used for gene expression analysis are listed in Additional file 1: Table S1 and were designed based on sequences available in GenBank (http://www.ncbi.nlm.nih.gov/entrez/query.fcgi). Analyses were performed with SYBR® Green chemistry (PerfeC_T_a SYBR® Green Supermix, ROX, Quanta BioSciences, 95055-500; Quanta BioSciences Inc., Gaithersburg, MD, USA) in a StepOnePlus™ Real-Time PCR System (Applied Biosystems Inc., Foster City, CA, USA). *Gapdh* and *Actb* were used in combination as housekeeping genes.

### Immunoblotting

Protein extracts from frozen TA cryosections [26], isolated mitochondria, or nuclear extracts were separated by SDS-PAGE and transferred to nitrocellulose membranes, which were blocked with 5% skim milk or 5% BSA solution. Membranes were incubated overnight at 4°C with the appropriate concentration of primary antibody (see Additional file 1: Table S2 for a full list of antibodies). Membranes were subsequently washed and incubated with the suitable HRP-conjugated secondary antibody for 1 h at room temperature and visualized with enhanced chemiluminescence. Quantification was performed with Image J Software (NIH, Bethesda, MD, USA), and values were normalized to the appropriate loading control.

### Single fiber immunofluorescence

Immunofluorescence staining was performed on isolated fixed EDL fibers [27] and imaged using a confocal microscope. Briefly, freshly excised EDL muscles were anchored at both ends and fixed with 2% paraformaldehyde in phosphate buffer for 1 h at room temperature. The muscles were then washed with PBS, kept in 50% glycerol at 4°C overnight, and were subsequently transferred to −20°C and stored until further use. The muscles were gradually transitioned through diminishing concentrations of glycerol, and individual fibers were then mechanically teased apart in a puddle of 0.04% saponin. The fibers were mounted onto glass slides and permeabilized with 0.2% Triton X-100 in 10% goat serum in PBS blocking solution. The fibers were then co-incubated overnight at 4°C with the appropriate primary antibodies (Additional file 1: Table S2) diluted in blocking solution. The fibers were washed with PBS and then co-incubated with the suitable fluorescent secondary antibodies for 2 h at room temperature. The fibers were subsequently washed three times with PBS, and DAPI was added to the first wash at a 0.5 μg/ml concentration in order to visualize the myonuclei. Glass cover slips were mounted onto the slides with DPX Mountant for histology (Fluka, 44581; Sigma-Aldrich, St. Louis, MO, USA) and sealed. Images were acquired with an Olympus Fluoview confocal microscope equipped with a × 60 objective (Olympus Corporation, Shinjuku, Tokyo, Japan).

### Cellular fractionation

Enriched mitochondrial and nuclear cellular subfractions were isolated by differential centrifugation, as previously described [24]. Briefly, the muscles were minced on ice and homogenized using a Teflon pestle and mortar and suspended in mitochondrial isolation buffer (MIB; 250 mM Sucrose, 20 mM HEPES, 10 mM KCl, 1.5 mM MgCl_2_, 1 mM EDTA, 1 mM EGTA) supplemented with protease (Complete, Roche, 1169749801; Roche Diagnostics, Basel, Switzerland) and phosphatase inhibitor cocktails (Cocktail 2 and 3, Sigma, P5726 and P0044). The homogenates were then centrifuged at 1,000 *g* for 10 min at 4°C to pellet the nuclei while mitochondrial and cytosolic fractions were contained within the supernate. The supernate fraction was re-centrifuged at 16,000 *g* for 20 min at 4°C to pellet the mitochondria. The mitochondrial pellet was washed twice and resuspended in a onefold dilution of MIB. Mitochondria were subsequently sonicated 3 × 3 s to yield the enriched mitochondrial fraction. Pellets containing nuclei were re-suspended in nuclear lysis buffer (1.5 mM MgCl_2_, 0.2 mM EDTA, 20 mM HEPES, 0.5 M NaCl, 20% glycerol, 1% Triton-X-100), incubated on ice for 30 min, and then sonicated 3 × 10 s followed by a final centrifugation step of 15 min at 16,000 *g*. The supernate was collected to obtain the enriched nuclear fraction. Protein concentrations within the samples were determined using the Bradford method. Fraction purity was determined by western blot analysis (Additional file 1: Figure S3).

### Statistics

Comparisons between WT and KO or TG and control (Con) and denervated (Den) animals were evaluated using two-way analyses of variance (ANOVA) on each of the treatment conditions. Bonferroni post-tests were performed when applicable. All values represent the mean ± SE. Data were considered statistically different if *P* < 0.05.

## Results

### Lack of PGC-1α results in diminished mitochondrial content, reduced muscle mass, and a myopathic phenotype

**Figure 1:**
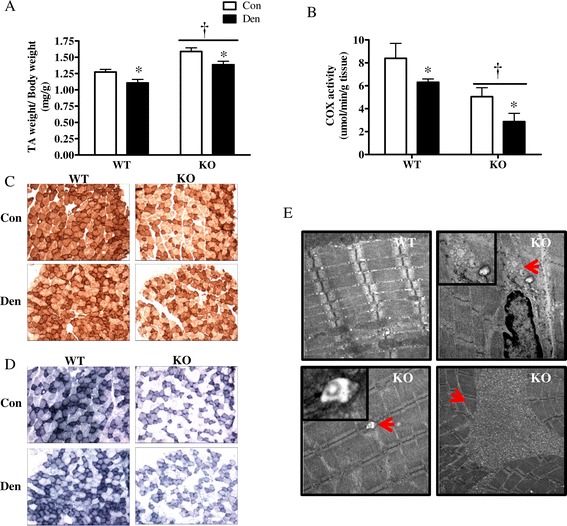
**PGC-1α KO animals have lower mitochondrial content and display myopathic features. (A)** TA muscle mass corrected for body weight. **(B)** Cytochrome C oxidase activity. **(C)** Representative images of COX staining of EDL muscle from Con and Den WT and KO animals. **(D)** Representative images of SDH staining of EDL muscle from Con and Den WT and KO animals. **(E)** EM images of control WT (top left panel) and PGC-1α KO muscle (top right and bottom two panels). PGC-1α KO animals display a myopathic phenotype characterized by the accumulation of multivesicular bodies (top right), aberrant mitochondria (bottom left) as well as tubular aggregates (bottom right). *P < 0.05, significant effect of denervation. †*P* < 0.05, significant effect of genotype (*n* = 4 to 8 for all groups). Con, control; COX, cytochrome C oxidase; Den, denervated; KO, knockout; TA, tibialis anterior; WT, wild type.

In order to ascertain the role of PGC-1α in skeletal muscle autophagy, 8-month-old whole-body PGC-1α KO and WT animals were unilaterally denervated for 7 days by severing the sciatic nerve of one hindlimb, with the contralateral limb serving as an internal Con. Denervation resulted in similar reductions in muscle mass in both WT and KO animals (Figure 1A). When corrected for body weight, the muscle mass of KO animals was greater than that of the WT littermates; however this was a result of their reduced body weight and not due to muscle hypertrophy, as fiber cross-sectional areas were smaller in mice lacking PGC-1α (Additional file 1: Figure S1A-C). Basal mitochondrial content was significantly diminished in KO animals, as evidenced from the reduced intensity of COX and SDH staining, as well as a lower COX activity measured biochemically (Figure 1B,C,D). Denervation resulted in 33% and 42% decrease in mitochondrial content in WT and KO mice, respectively (Figure 1B). Electron microscopy images revealed myopathic features in PGC-1α KO mice, evident from the accumulation of multivesicular bodies, aberrant mitochondria as well as tubular aggregates (Figure 1E). These features are suggestive of autophagic deficiency in animals lacking PGC-1α.

### Attenuated autophagic signaling, lower lysosomal abundance, and decreased denervation-induced autophagy flux in mice lacking PGC-1α

**Figure 2:**
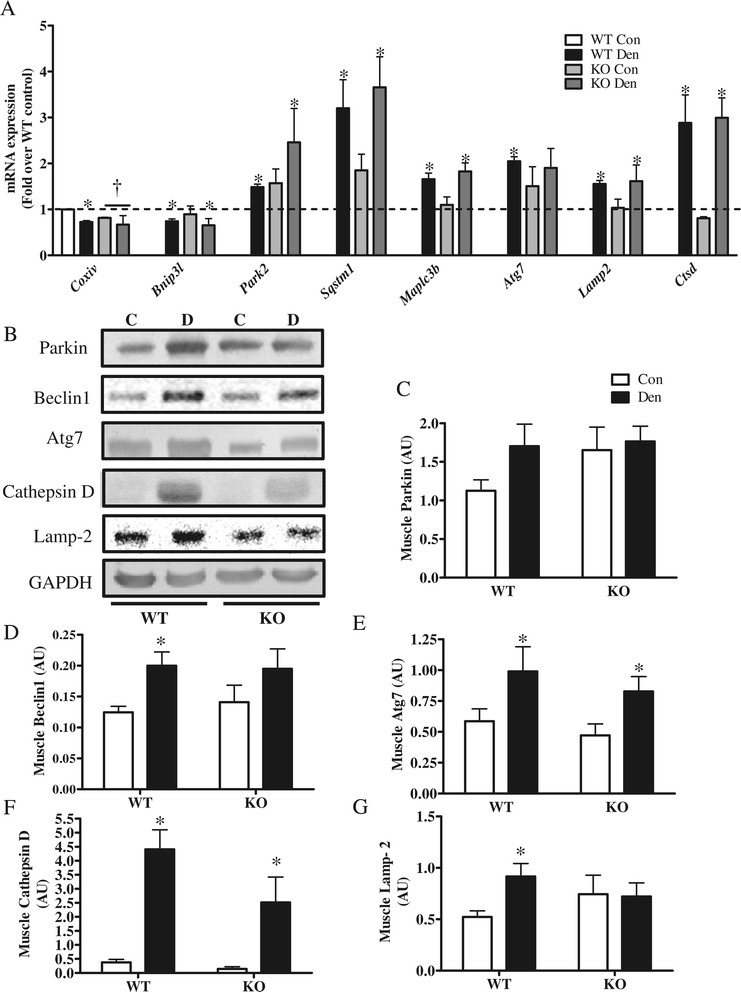
**Lack of PGC-1α results in attenuation of autophagic signaling induced by denervation. (A)** Autophagy gene expression measured by real time PCR. mRNA fold change between wild type (WT) and PGC-1α KO (KO) control (Con) and denervated (Den). All groups were compared to WT Con, and *Gapdh* and *Actb* were used as housekeeping genes. **(B-G)** Blots and quantification of autophagic proteins in control (C; Con), denervated (D; Den) WT and KO animals. **(B)** Representative blots. Quantification of **(C)** parkin, **(D)** Beclin 1, **(E)** Atg7, **(F)** Cathepsin D and **(G)** Lamp-2. *P < 0.05 significant difference between Con and Den. GAPDH was used as a loading control (*n* = 4 to 8 for all groups). AU, Arbitrary units; GAPDH, glyceraldehyde 3-phosphate dehydrogenase.

We next set out to assess the role of the co-activator in the transcriptional regulation of key autophagy genes and in autophagy signaling, both basally and in response to denervation. Denervation resulted in increased expression of various genes that are involved in a variety of aspects of autophagy and mitophagy. Significant increases in *Park2*, *Sqstm1*, *Maplc3b*, *Atg7*, *Lamp2*, and *Ctsd* mRNA expression in response to denervation (Figure 2A) were observed. We did not find a significant difference in the mRNA expression of autophagic factors between WT and KO animals, other than an attenuated denervation-induced increase in *Atg7.* We also assessed autophagy signaling using western blot analysis. In accordance with our mRNA data, we found significant increases in autophagy proteins in response to denervation (Figure 2B). Beclin1, Atg7, Cathepsin D, and lysosome-associated membrane protein 2 (Lamp-2) all increased, while parkin tended to increase in WT animals (Figure 2B,C,D,E,F,G). Although there was no significant difference in the content of autophagy proteins between WT and KO animals basally, the denervation-induced increases in Beclin1 and Lamp-2 levels were significantly attenuated in KO muscle, while Cathepsin D induction tended to be lower in KO animals.

**Figure 3:**
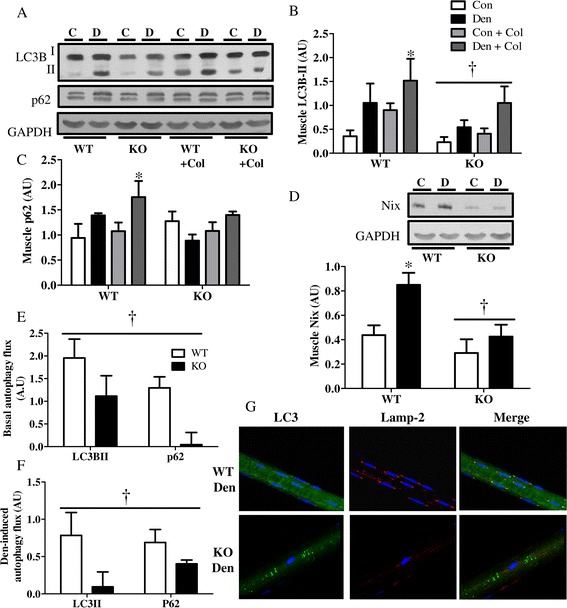
**Lack of PGC-1α results in reduced lysososmal abundance and denervation-induced autophagy flux. (A-C)** Blots and quantification of autophagic proteins in control (Con), denervated (Den) WT, and PGC-1α KO (KO) animals; treated with vehicle (water) or 0.4mg/kg/day colchicine (col) for 4 days. **(A)** Representative blots. Quantification of **(B)** LC3BII and **(C)** p62, **(D)** Representative blot and quantification of Nix, **(E)** basal autophagy flux, and **(F)** denervation-induced autophagy flux, **(G)** Confocal images of fixed single fibers immuno-stained for LC3 (green) and lysosomal Lamp-2 (red) and colocalization is shown in yellow (Merge) and represents autophagosomes within lysosomes. Nuclei are in blue (DAPI). *P < 0.05 significant difference between Con and Den. †*P* < 0.05 significant effect of genotype. GAPDH was used as a loading control (*n* = 3 to 5 for all groups). AU, arbitrary units; GAPDH, glyceraldehyde 3-phosphate dehydrogenase; KO, knockout; WT, wild type.

In order to ascertain the influence of PGC-1α on autophagy flux, we treated the animals with the microtubule de-stabilizing drug colchicine, previously reported to effectively block autophagic degradation [22] (Figure 3). Denervation resulted in increased accumulation of LC3B-II, p62, and Nix (Bnip3l) proteins in WT muscle. Colchicine treatment resulted in further accumulations of LC3B-II and p62 in denervated WT muscle, indicating a successful block in autophagic degradation with the drug (Figure 3A,B,C). In contrast, PGC-1α KO animals exhibited an attenuated LC3B-lipidation and expression of the mitophagy-specific receptor Nix both basally and in response to denervation (Figure 3A,B,D). No significant difference in p62 expression was observed basally between the genotypes, and p62 levels did not change in KO animals with either Den or Col treatment (Figure 3C). Importantly, both basal and denervation-induced p62 and LC3B-II flux were lower in KO animals, as evidenced by a smaller accumulation of LC3BII and p62 in KO animals treated with colchicine (Figure 3E,F). We further examined lysosomal abundance and autophagy flux in KO and WT animals using confocal microscopy (Figure 3G). We did this by isolating single fibers from fixed EDL muscles and co-immunostaining them for LC3B and Lamp-2. KO animals had lower lysosomal abundance, as indicated by a diminished intensity and frequency of red fluorescence, and this was especially evident following denervation (Figure 3G and Additional file 1: Figure S2). We also noted an increase in the colocalization (yellow) of autophagosomes and lysosomes and their aggregation in the perinuclear region of denervated WT muscles (Figure 3G, Merge). This was not as evidenced in the denervated KO muscle, as indicated by decreased yellow flourescence. Taken together, these results indicate that the lack of PGC-1α results in lower lysosomal abundance and reduced autophagy flux in response to denervation.

### Mitophagy is attenuated in PGC-1α-deficient muscle

**Figure 4:**
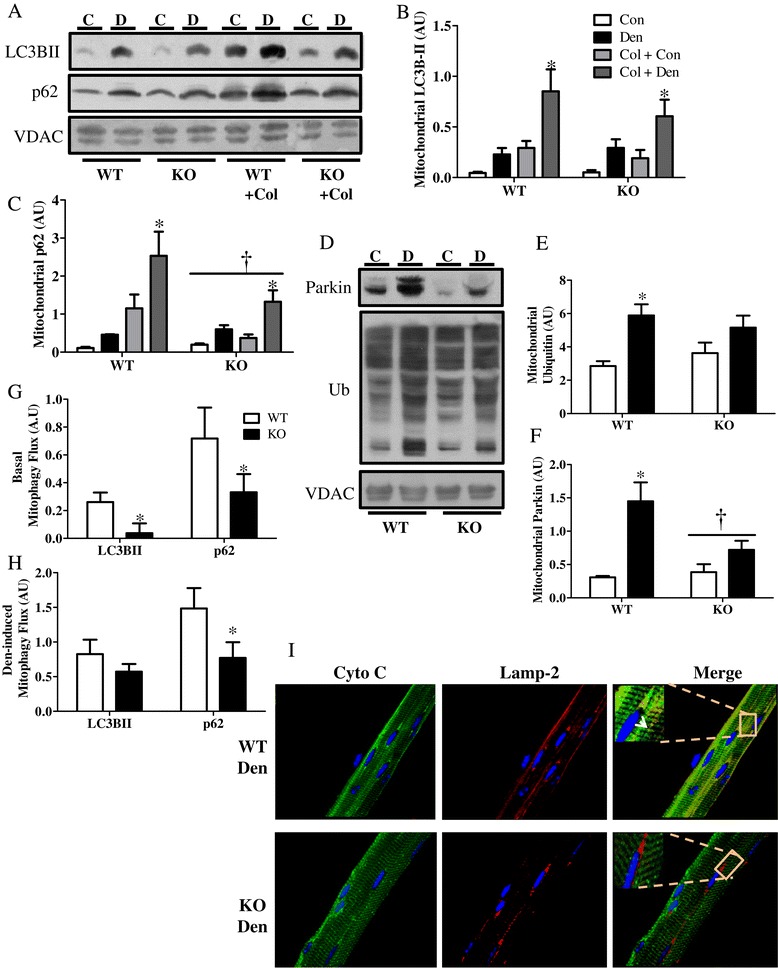
**Lack of PGC-1α results in attenuated mitophagy flux. (A-D)** Blots and quantification of autophagic proteins in isolated mitochondrial fractions in control (Con), denervated (Den) WT, and PGC-1α KO (KO) animals treated with vehicle (water) or colchicine (col) 0.4mg/kg/day for 4 days. **(A)** Representative blots. Quantification of **(B)** LC3BII and **(C)** p62. **(D)** Representative blots. Quantification of mitochondrial **(E)** ubiquitin, **(F)** parkin, **(G)** basal mitophagy flux, and **(H)** denervation-induced mitophagy flux. **(I)** Confocal images of fixed single fibers immuno-stained for cytochrome c (as a mitochondrial marker, green) and lysosomal Lamp-2 (red); their colocalization (yellow) represents mitochondria within lysosomes. *P < 0.05 significant difference between Con and Den. †*P* < 0.05 significant effect of genotype. VDAC was used as loading control (*n* = 3 to 5 for all groups). AU, arbitrary units; KO, knockout; VDAC, voltage-dependent anion channel; WT, wild type.

Since PGC-1α is a major metabolic regulator that plays a key role in mitochondrial homeostasis, we were interested in elucidating the role of this co-activator in mitochondrial removal by mitophagy. During denervation, the mitochondrial pool within skeletal muscle undergoes a substantial reduction (33% to 42%, Figure 1B). To determine the involvement of PGC-1α in mitophagy, we isolated mitochondria from both the denervated and control gastrocnemius muscle of WT and KO animals that were treated with either vehicle or colchicine (Figure 4). Denervation resulted in an increase in the localization of LC3BII, p62, and parkin to the mitochondria, as well as an elevated overall ubiquitination of mitochondrial substrates (Figure 4A,B,C,D,E), culminating in increased mitophagy flux (Figure 4H). These findings indicate that mitophagy is involved in the removal of mitochondria from the muscle basally and during denervation. This response was reduced in the KO animals (Figure 4G,H,I). The muscle from KO animals exhibited similar basal localization of autophagic proteins to the mitochondria (Figure 4A,B,C,D,E,F) but had an attenuated increase in mitochondrial localization of these proteins with denervation. Importantly, KO animals exhibited lower basal and denervation-induced mitophagy flux (Figure 4G,H). To further investigate mitophagy flux, we co-stained the isolated single EDL fibers with mitochondrial cytochrome c (Cyto C; green) as well as with Lamp-2 (red) and assessed their co-localization (Merge; yellow) using confocal microscopy (Figure 4I). The images suggest a decrease in the co-localization of mitochondria with lysosomes in denervated KO animals compared to WT animals, further supporting a decrease in mitophagy flux in the KO animals in response to denervation.

### TFEB protein levels are induced with denervation and may mediate PGC-1α action on autophagy

**Figure 5:**
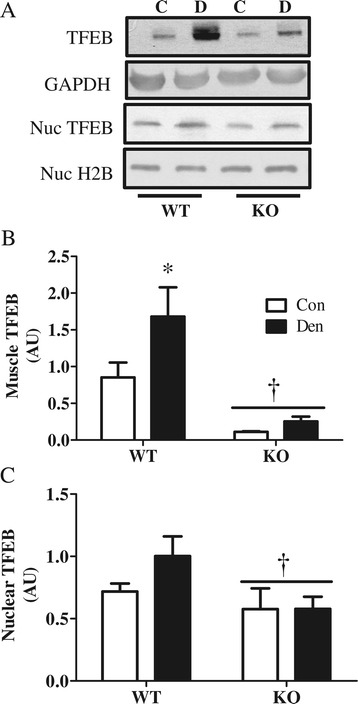
**Lack of PGC-1α results in lower TFEB protein levels and nuclear localization. (A-C)** Blots and quantification TFEB protein levels between wild type (WT) and PGC-1α KO (KO) control (Con) and denervated (Den). **(A)** Representative blots. Quantification of **(B)** whole muscle TFEB protein. **(C)** Nuclear localization of TFEB in TA muscle. *P < 0.05 significant difference between Con and Den. †*P* < 0.05 significant effect of genotype. GAPDH was used as loading control for whole muscle, and histone 2 B (H2B) was used as nuclear loading control (*n* = 4 to 8 for all groups). GAPDH, glyceraldehyde 3-phosphate dehydrogenase; Nuc. H2B, nuclear histone 2B; Nuc. TFEB, nuclear transcription factor EB; TFEB, nuclear transcription factor EB.

To examine the role of PGC-1α in the expression of autophagy genes, we examined TFEB, the master transcriptional regulator of the autophagy-lysosome system, as several recent studies have indicated an interplay between these two factors [16,17,28,29]. TFEB protein levels were increased with denervation in WT animals (Figure 5A,B,C), and this tended to impact the levels of TFEB in the nucleus. In the KO animals, TFEB levels and nuclear localization were lower under basal conditions (*P* < 0.05) and did not change in response to denervation (Figure 5A,B,C).

### Overexpression of PGC-1α results in increased mitochondrial content and protection from denervation-induced mitochondrial loss and muscle atrophy

**Figure 6:**
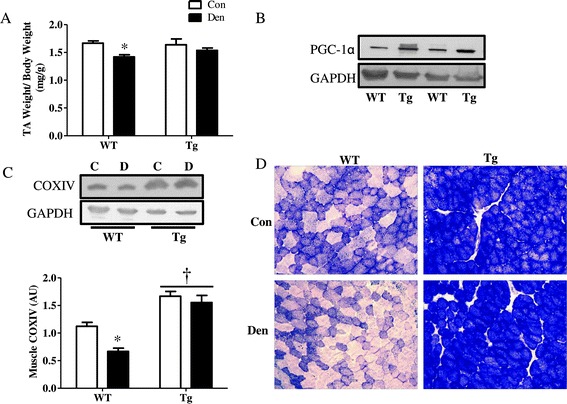
**PGC-1α over-expressing animals have higher mitochondrial content and are protected from denervation-induced muscle atrophy. (A)** TA muscle mass corrected for body weight. **(B)** PGC-1α blot confirming overexpression in TA muscle. **(C)** Representative blot and quantification of COXIV as a mitochondrial content marker. **(D)** Representative images of SDH staining of TA muscle from Con and Den WT and Tg animals. *P < 0.05, significant effect of denervation. †*P* < 0.05, significant effect of genotype (*n* = 3 to 5 for all groups). Con, control; Den, denervated; GAPDH, glyceraldehyde 3-phosphate dehydrogenase; PGC-1α, peroxisome proliferator co-activator 1 alpha; TA, tibialis anterior; Tg, transgenic; WT, wild type.

Since our results indicate a potential role for PGC-1α in regulating autophagy flux in response to denervation, we investigated whether PGC-1α alone was sufficient to induce autophagy. Thus, we compared WT to muscle-specific PGC-1α overexpressing animals (Tg). TA muscle from Tg animals displayed a much more oxidative phenotype (Figure 6C,D), and they were protected from denervation-induced muscle atrophy (Figure 6A). We confirmed PGC-1α over-expression in Tg animals by immunoblotting for PGC-1α (Figure 6B) and COXIV (Figure 6C), a downstream target of the co-activator, as well as with histochemical staining for SDH (Figure 6D). The TA muscles of Tg animals were significantly richer in mitochondria and exhibited a reduced loss of mitochondrial content in response to denervation, as indicated by greater COXIV protein (Figure 6C) and darker SDH staining (Figure 6D), compared to WT controls.

### PGC-1α overexpression increases lysosomal and mitophagy receptor expression

**Figure 7:**
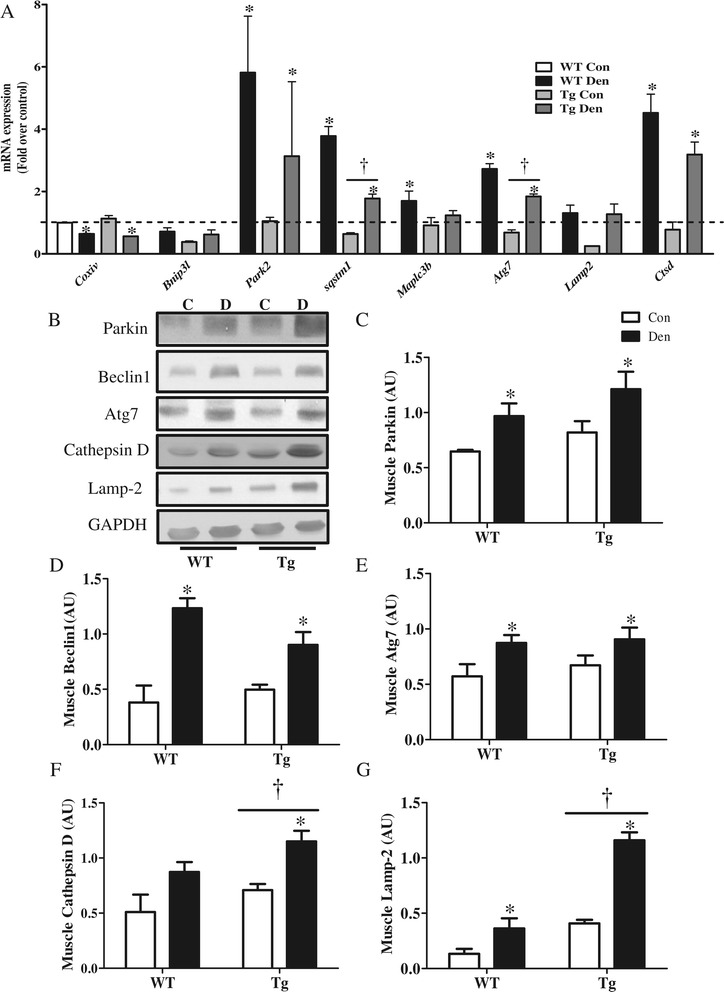
**Over-expression of PGC-1α results in enhanced lysosomal protein expression induced by denervation. (A)** Autophagy gene expression. mRNA fold changes between wild type (WT) and PGC-1α Tg (Tg) control (Con) and denervated (Den). All groups are compared to WT con, *Gapdh* and *Actb* were used as housekeeping genes. **(B-G)** Blots and quantification of autophagic proteins in Con, Den WT and Tg animals. **(B)** Representative blots. Quantification of **(C)** Parkin, **(D)** Beclin 1, **(E)** Atg7, **(F)** Cathepsin D, and **(G)** Lamp-2. *P < 0.05 significant difference between Con and Den. †*P* < 0.05, significant effect of genotype. GAPDH was used as a loading control (*n* = 3 to 5 for all groups). AU, arbitrary units; GAPDH, glyceraldehyde 3-phosphate dehydrogenase.

**Figure 8:**
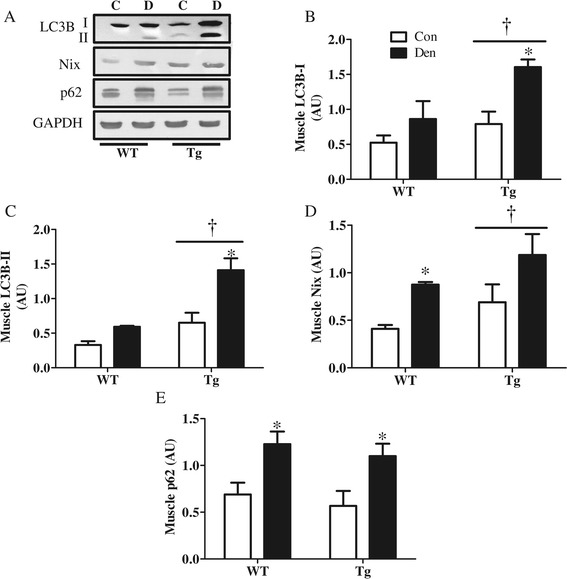
**Elevated PGC-1α results in greater basal and denervation-induced autophagy protein expression. (A-E)** Blots and quantification of autophagic proteins in control (Con), denervated (Den) WT and PGC-1α Tg (Tg) animals. **(A)** Representative blots. Quantification of **(B)** LC3BI, **(C)** LC3BII, **(D)** Nix, and **(E)** p62. *P < 0.05 significant difference between Con and Den. †*P* < 0.05 significant effect of genotype. GAPDH was used as a loading control (*n* = 3 to 5 for all groups). AU, arbitrary units; GAPDH, glyceraldehyde 3-phosphate dehydrogenase.

We examined the effect of PGC-1α overexpression on the levels of autophagy genes and proteins basally and in response to denervation. Real-time PCR analyses confirmed that 7 days of denervation resulted in increased expression of various autophagy and mitophagy genes, such as *Park2*, *Sqstm1*, *Maplc3b*, *Atg7*, and *Ctsd* (Figure 7A) consistent with our earlier results (Figure 2A). We found a decrease (*P* < 0.05) in the basal expression of *Sqstm1*, *Bnip3l*, and *Atg7* in Tg animals. In addition, we also observed attenuated denervation-induced increases in *Sqstm1*, *Maplc3b*, and *Atg7.* We also assessed autophagy signaling by western blot analysis. Parkin, Beclin1, and Atg7 protein levels were similar in WT and Tg animals, both basally and in response to denervation (Figure 7B,C,D,E). However, the levels of the lysosomal proteins Cathepsin D and Lamp-2 were significantly higher in Tg as compared to WT animals, and these were markedly increased in the denervated Tg muscle (Figure 7F,G). Tg animals also displayed significantly higher levels of both LC3I and II than WT controls, and these were augmented to a greater extent by denervation compared to WT animals (Figure 8A,B,C). Similarly, Nix protein content was higher in Tg, as compared to WT animals, both basally and in response to denervation (Figure 8D), while p62 levels were similar in both genotypes (Figure 8E). Taken together, these data suggest that PGC-1α selectively induces the expression of specific autophagy and lysosomal proteins.

### PGC-1α overexpression resulted in reduced autophagy markers in the mitochondrial subfraction

**Figure 9:**
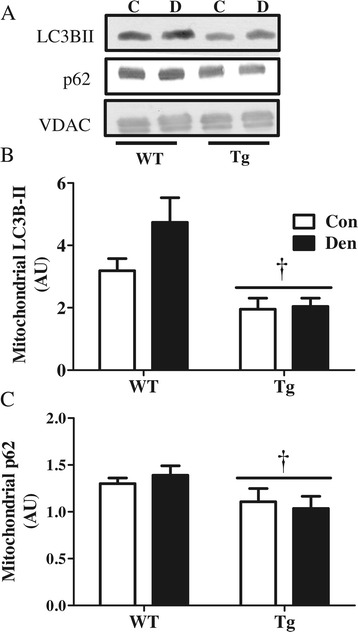
**PGC-1α Tg animals demonstrate lower presence of autophagy markers in isolated mitochondria. (A-C)** Blots and quantification of autophagic proteins on isolated mitochondria in control (Con), denervated (Den) WT and PGC-1α Tg (Tg). **(A)** Representative blots. Quantification of **(B)** LC3BII and **(C)** p62. †*P* < 0.05 significant effect of genotype. VDAC was used as loading control (*n* = 3 for all groups). AU, arbitrary units; VDAC, voltage-dependent anion channel.

Since we found a protective effect of PGC-1α overexpression on mitochondria following denervation (Figure 6), we wanted to investigate whether mitophagy was affected. Our results indicate that PGC-1α overexpression leads to a decreased localization of LC3BII and p62 to the mitochondrial fraction in Tg muscle, both basally and in response to denervation (Figure 9A,B,C).

### TFEB is induced with denervation and may mediate PGC-1α action on autophagy

**Figure 10:**
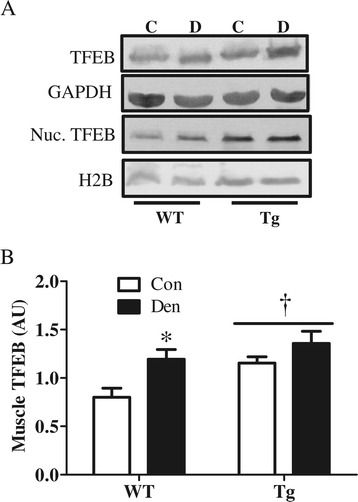
**Elevated PGC-1α results in increased TFEB protein levels. (A-B)** Blots and quantification TFEB protein level in TA muscle and in nuclear fraction of wild type (WT) and PGC-1α Tg (Tg) control (Con) and denervated (Den). **(A)** Representative blots. **(B)** Quantification of TFEB protein in TA muscle. *P < 0.05 significant difference between Con and Den. †*P* < 0.05 significant effect of genotype. GAPDH was used as loading control for whole muscle and histone 2 B (H2B) was used as nuclear loading control (*n* = 3 to 5 for all groups). GAPDH, glyceraldehyde 3-phosphate dehydrogenase. H2B, histone 2 B; Nuc. TFEB, nuclear transcription factor EB; TFEB, transcription factor EB.

Since we found a decrease in TFEB protein levels in animals lacking PGC-1α (Figure 5), we further investigated the effects of PGC-1α overexpression on this transcription factor. TFEB protein levels were significantly higher in PGC-1α Tg animals (Figure 10A,B). Similar to our earlier findings, TFEB increased with denervation in WT animals, to a level that was similar to the higher basal level evident in Tg animals (Figure 10A,B). TFEB protein was not further induced by denervation in Tg animals.

## Discussion

Metabolic plasticity is a unique property which permits the fine tuning of energy production to meet energy demands in skeletal muscle, allowing for adaptations in response to alterations in nutrient availability, hormonal stimuli, and contractile activity. This property makes muscle a pillar of whole-body homeostasis, in particular during energetic distress. Interestingly, both the autophagy-lysosome system and the transcriptional co-activator PGC-1α have been separately documented to contribute to whole-body metabolic homeostasis, as well as to muscle plasticity, in response to alterations in nutrient availability and contractile activity [10,15,29-34]. However, the role of PGC-1α in autophagy and mitophagy has not been dissected thus far. In this study, we illuminate a role for PGC-1α in autophagy and mitophagy in skeletal muscle in response to chronic muscle disuse. Moreover, we identify the transcription factor TFEB to be a potential target of PGC-1α in the regulation of autophagy in this tissue.

Our results have confirmed the myopathic phenotype evident in muscles of animals lacking PGC-1α. The muscle of KO animals was characterized by a diminished mitochondrial content, smaller cross-sectional area, and an accumulation of damaged organelles and multivesicular bodies as evidenced by the appearance of abnormal structures in EM images. Some of the myopathic features of PGC-1α KO muscle are reminiscent of those found in autophagy-deficient animals which also exhibit deficient mitochondria and increased apoptosis [10,35,36]. We did not note a basal difference between WT and KO animals in the mRNA or protein expression of various autophagy markers. However, it is possible that an earlier time point following the onset of denervation, when autophagy gene expression may have peaked, could have revealed some endogenous expression differences between the two genotypes in response to this muscle atrophy stimulus. Importantly, we did observe an attenuated induction in LC3B lipidation and protein expression of lysosomal factor Lamp-2 with denervation in KO animals. A lack of PGC-1α also resulted in reduced basal as well as denervation-induced autophagy flux, suggesting that the presence of PGC-1α has a significant impact on the maintenance of autophagy in muscle.

In contrast to the mitochondrial phenotype observed in KO animals, over-expression of the co-activator resulted in a highly oxidative muscle that was protected from denervation-induced loss of mitochondria and muscle mass. This was evident from the much darker SDH staining, as well as the increased COXIV protein expression in Tg animals that did not decrease with denervation. This protection has been previously documented to be a result of improved mitochondrial function [37], cellular oxidative status [38], and suppression of FoxO3-mediated catabolism [13]. Similar to our observations with KO animals, overexpression of the coactivator did not result in dramatic alterations in autophagy protein expression. However, levels of the lysosomal marker Lamp-2 and the protease cathepsin D were significantly induced in Tg animals and were further increased with denervation. Moreover, LC3B protein levels, as well as LC3B lipidation were both enhanced in the Tg animals, suggesting an increased autophagy flux mediated by PGC-1α. In another study involving the use of fasting as an inducer of autophagy, we have confirmed the increase in autophagy flux in Tg animals (Additional file 1: Figure S4).

To investigate the role of PGC-1α in mitophagy, we examined the expression of the mitophagy receptor Nix (Bnip3L) as well as the localization of autophagy factors to isolated mitochondria. The expression of Nix was significantly reduced in KO, and strongly induced in Tg, when compared to WT animals, indicating that Nix may be under the control of PGC-1α. This could be mediated by the PGC-1α-HIF-1α axis, as Nix was found to be under HIF-1α control during hypoxia [39], and HIF-1α was documented to be stabilized by PGC-1α in muscle cells [40]. However, further research is required to confirm this interaction in muscle. We also noted an enhanced localization of LC3B-II, p62, parkin as well as ubiquitin to isolated mitochondria with denervation in WT animals, but this effect was attenuated in KO animals. Indeed, both basal and denervation-induced mitophagy flux were reduced, indicating impaired mitophagy in the absence of PGC-1α. We have previously documented oxygen consumption deficits and enhanced susceptibility to apoptosis in mitochondria of the PGC-1α KO muscle [18]. These findings can now likely be attributed to deficient mitochondrial turnover, resulting from a combination of an impairment in mitochondrial biogenesis [1,19,41] as well as mitophagy in the absence of PGC-1α.

**Figure 11:**
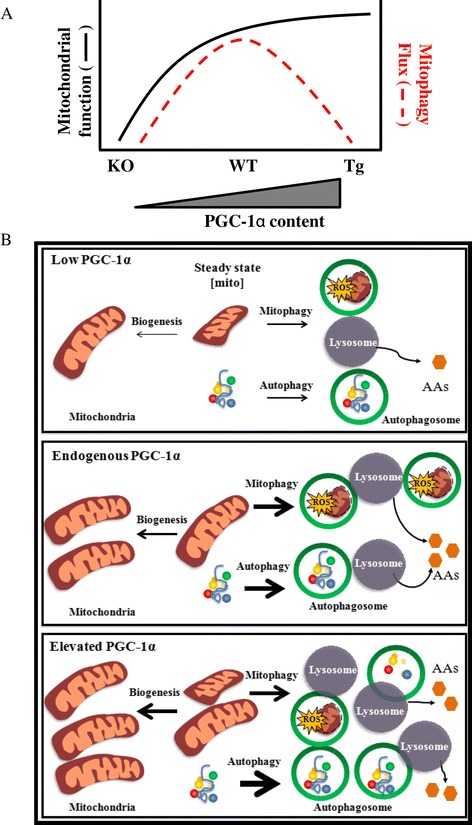
**Proposed relationship between PGC-1α expression, mitochondrial function, and mitophagy during cellular stress. (A-B)** Upon cellular metabolic stress such as denervation or nutrient deprivation lack of PGC-1α results in diminished mitochondrial function, biogenesis, and impaired autophagy and mitophagy, whereas its overexpression results in superior mitochondrial function and biogenesis as well as enhanced autophagy, but reduced mitophagy. **(A)** Hypothetical graphical representation of the relationship between levels of PGC-1α, mitochondrial function, and mitophagy. **(B)** Schematic outlining mitochondrial turnover during denervation-induced metabolic stress in muscle in light of variations in PGC-1α expression. Steady state mitochondrial content is represented in the center of each panel. In the presence of endogenous PGC-1α levels (middle panel), biogenesis is active at a low level during denervation, but both mitophagy and autophagy flux are enhanced leading to a reduced steady state mitochondrial content. When the expression of PGC-1α is abolished (upper panel), biogenesis is further reduced during denervation, as are autophagy and mitophagy flux, leading to a smaller, dysfunctional pool of mitochondria. When PGC-1α levels are elevated (lower panel), biogenesis is higher while mitophagy is lower than normal during denervation, leading to a maintained organelle content. Arrow thickness provides an indication of the magnitude of the pathway. AAs, amino acids; PGC-1α, peroxisome proliferator co-activator 1 alpha; ROS, reactive oxygen species.

Interestingly, we also noted reduced localization of autophagic markers p62 and LC3B-II to the mitochondrial fraction of Tg animals, both basally and in response to denervation. This corresponded to the enhanced protection of mitochondrial content during denervation in these animals, and it occurred despite increases in LC3B and the mitophagy receptor Nix in whole muscle extracts. We also confirmed reduced mitophagy flux under fasting conditions in PGC-1α Tg animals compared to WT littermates (Additional file 1: Figure S5). These data suggest that PGC-1α mediates a reduced targeting of mitochondria for mitophagy when mitochondrial function and content are high. Based on our data, we contend that physiological levels of PGC-1α are required for the proper propagation of mitophagy in muscle, as a lack of PGC-1α results in reduced mitochondrial turnover, culminating in the accumulation of defective mitochondria, increased susceptibility to cell death [18] and overall muscle atrophy. In contrast, PGC-1α overexpression is protective of mitochondrial mass via several mechanisms (Figure 11A,B). First, PGC-1α has been shown to improve mitochondrial function [42,43] and enhance cellular antioxidant capability [38] which can result in reduced mitochondrial targeting for degradation. Second, mitochondrial fragmentation is required for mitophagy to occur, and PGC-1α augments the expression of factors involved in mitochondrial fusion such as mitofusin 1 and 2 [44], resulting in a more reticular mitochondrial phenotype that can better evade mitophagy. Third, PGC-1α overexpression has been documented to block FoxO3-mediated transcriptional activity [13], and since FoxO3 drives the expression of multiple mitophagy factors such as Mul1 and Bnip3 [45,46], repression of FoxO3activity may be sufficient to protect mitochondria from elimination.

The involvement of autophagy during denervation has recently been brought into question. Some evidence has indicated a block in autophagy early in denervation [47,48], while other data point to the contrary at later time points [10,21,22,46,49-51]. Here, we demonstrate that autophagy and mitophagy flux were both elevated at 7 days of denervation, with mitophagy contributing to mitochondrial loss during disuse. Indeed, localization of LC3B-II, p62, and parkin to the mitochondria were all induced during denervation resulting in enhanced mitophagy flux in WT animals.

Our study has also revealed key evidence supporting a role for PGC-1α in lysosomal biogenesis. Lack of PGC-1α resulted in reduced denervation-induced lysosomal protein expression and overall lysosomal abundance, while PGC-1α overexpression provoked an increase in basal and denervation-induced levels of the lysosomal proteins Lamp-2 and cathepsin D. Furthermore, we found a correlation (Pearson *r* = 0.84; data not shown) between levels of PGC-1α and the lysosomal master regulator TFEB. This further supports findings by Scott *et al.* [17] on the coordinated regulation of PGC-1α and TFEB, which works to ensure the proper matching between mitochondrial removal and biogenesis. Thus, we highlight a role for PGC-1α in lysosomal biogenesis which could be mediated, at least in part, by TFEB.

## Conclusions

Our results suggest a role for the transcriptional co-activator PGC-1α in the regulation of autophagy-lysosomal machinery and mitophagy in skeletal muscle. A lack of PGC-1α results in reduced disuse-induced autophagy and mitophagy signaling and flux, whereas PGC-1α overexpression increased lysosomal abundance and bulk autophagy flux while suppressing mitophagy (Figure 11B) Therefore, our findings elucidate a previously unidentified role for PGC-1α in the fine tuning of autophagy and mitophagy in skeletal muscle, and the identification of pharmacological targets along the PGC-1α-autophagy axis could be of therapeutic benefit to those suffering from metabolic or muscle wasting myopathies.

## Additional file

Additional file 1:
**Table S1.** Additional information and data supporting the manuscript by Vainshtein *et al*. Primers used for gene transcript analysis. **Table S2.** Antibodies used. **Figure S1.** PGC-1α KO animals muscle cross-sectional area. **Figure S2.** Confocal images of immuno-stained fixed single fibers from WT and PGC-1α KO animals. **Figure S3.** Confirmation of fraction purity by representative western blots. **Figure S4.** Autophagy protein expression and flux in PGC-1α Tg animals with 24 h fasting. **Figure S5.** Mitophagy flux in PGC-1α Tg animals with 24 h fasting.

## Abbreviations

Atg7: Autophagy related 7
Con: Control
COX: Cytochrome oxidase
Den: Denervated
EDL: Extensor digitorum longus
FoxO3: Forkhead box O3
KO: Knockout
Lamp-2: Lysosome-associated membrane protein 2
LC3/Maplc3: Microtubule-associated protein 1 light chain 3
MCK: Muscle creatine kinase
Nix/Bnip3l: Bcl-2/adenovirusE1B 19 kDa interacting protein 3-like
p62/Sqstm1: Sequestosome 1
Parkin/park2: RBR E3 ubiquitin protein ligase
PGC-1α: Peroxisome proliferator co-activator 1 alpha
ROS: Reactive oxygen species
SDH: Succinate dehydrogenase
TA: Tibialis anterior
TFEB: Transcription factor EB
Tg: Transgenic
WT: Wild type
