# Supplementary material for: PGC-1α modulates denervation-induced mitophagy in skeletal muscle
Source: Skelet Muscle. 2015 Mar 18;5:9. doi: 10.1186/s13395-015-0033-y (PMC4381453; doi:10.1186/s13395-015-0033-y)
Supplement: Additional file 1: — Table S1. Additional information and data supporting the manuscript by Vainshtein et al. Primers used for gene transcript analysis. Table S2. Antibodies used. Figure S1. PGC-1α KO animals muscle cross-sectional area. Figure S2. Confocal images of immuno-stained fixed single fibers from WT and PGC-1α KO animals. Figure S3. Confirmation of fraction purity by representative western blots. Figure S4. Autophagy protein expression and flux in PGC-1α Tg animals with 24 h fasting. Figure S5. Mitophagy flux in PGC-1α Tg animals with 24 h fasting. [file 13395_2015_33_MOESM1_ESM.pdf]

Supplementary Data for:

PGC-1α contributes to denervation-induced mitophagy in skeletal muscle

Anna Vainshtein, Eric M. Desjardins, Andrea Armani, Marco Sandri, and David A. Hood

Fig.S1

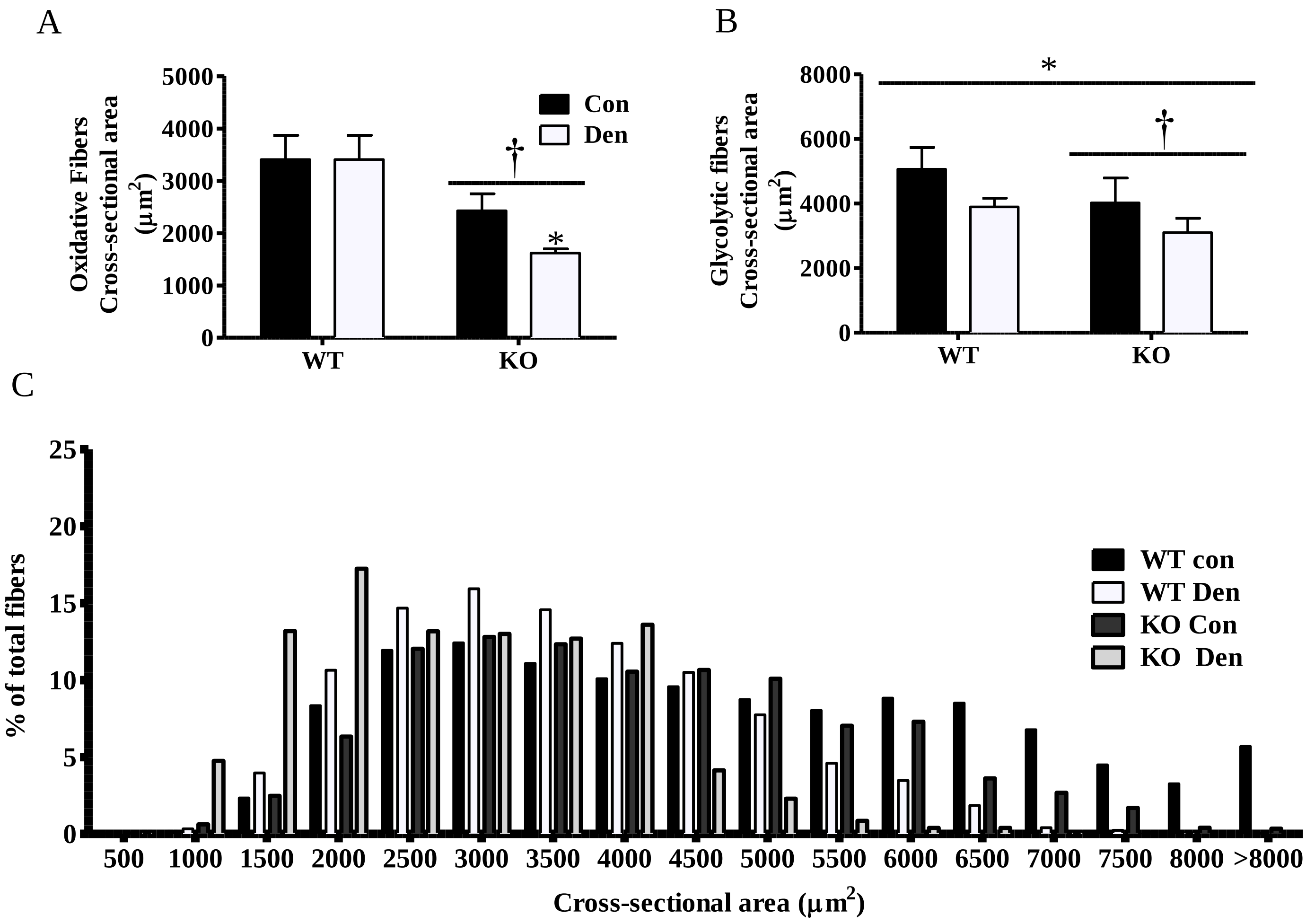

Fig.S1 PGC-1α KO animals have lower muscle cross-sectional area . A-C. TA muscle fiber cross sectional area of WT KO Con and Den was measured from serial cross sections stained with SDH. A. Average size of oxidative fibers. B. Average size of glycolytic fibers. C. Fiber size distribution . *P<0.05, significant effect of denervation .† P<0.05, significant effect of genotype (n=4 for all groups).

Fig.S2

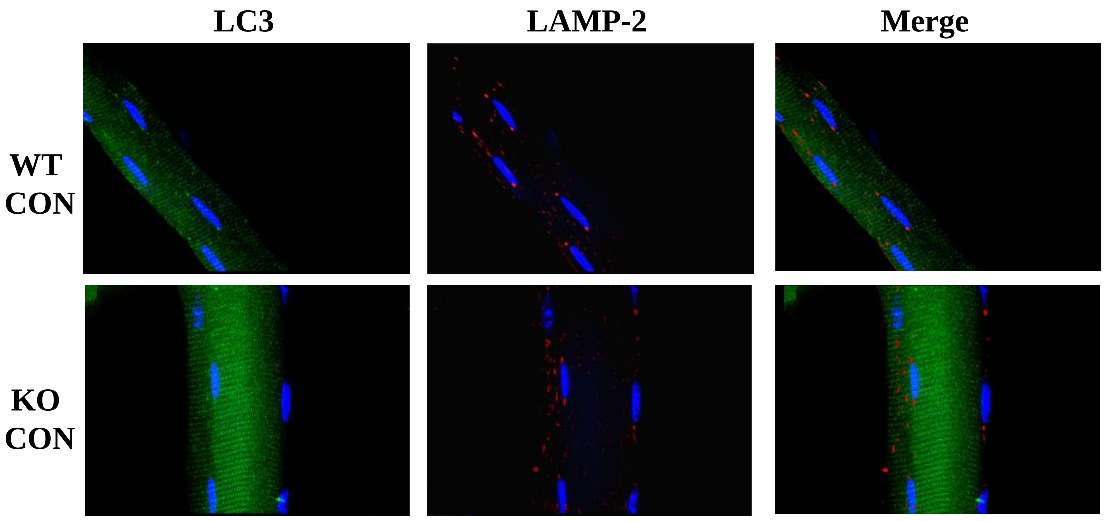

Fig.S2 Confocal images of fixed single fibers immuno-stained for LC3 (green) and lysosomal Lamp-2 (red). Colocalization is shown in yellow (Merge) which represents autophagosomes within lysosomes. Nuclei are in blue (Dapi).

Fig.S3

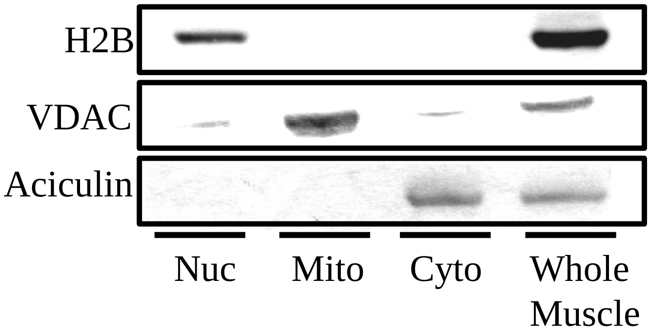

Fig S3. Fraction purity. Representative blots demonstrating the quality of cellular fractionation in nuclear (Nuc), mitochondrial (Mito), cytosolic (Cyto) cellular subfractions as well as whole muscle lysates, for positive control. H2B is a nuclear factor, VDAC is a mitochondrial protein and Aciculin is a cytosolic protein. Equal amounts of protein were loaded for each.

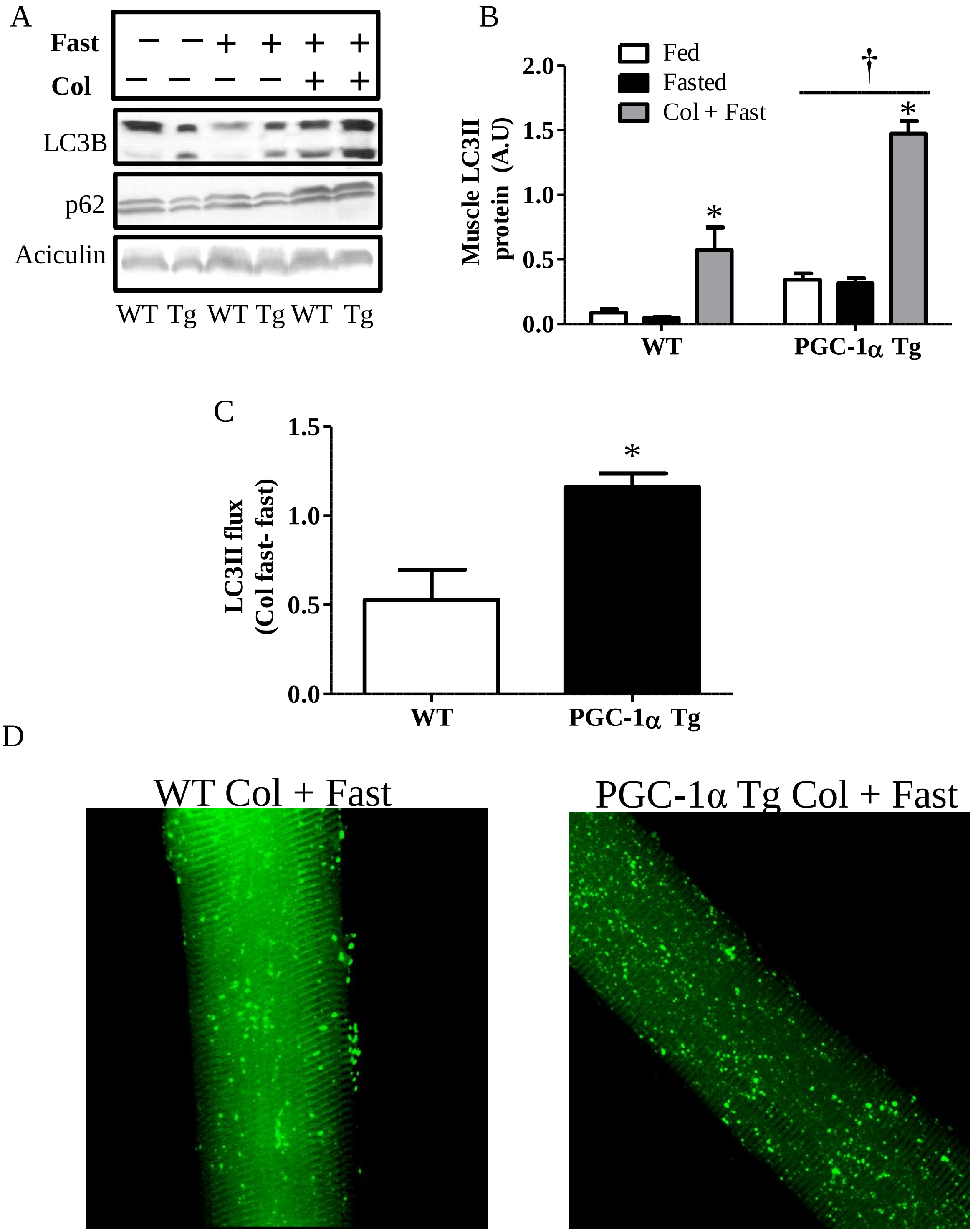

Fig. S4: Autophagy protein expression and flux. A) Representative blots of autophagic proteins and B-C) quantification of protein expression and autophagy flux in WT and PGC-1αTg animals that were fed (Control) or fasted (24h), with or without colchicine (Col) treatment; B) LC3II; C) autophagy flux. GAPDH was used as loading control. D) Representative images of single fibers isolated from FDB muscle transfected with LC3-YFP (Green) showing increased fasting-induced autophagsome accumulation in PGC-1αTg treated with colchicine as compared to WT. *P<0.05 significant effect of treatment. †P<0.05 significant effect of genotype. Aciculin was used as a loading control (n=3 for all groups).

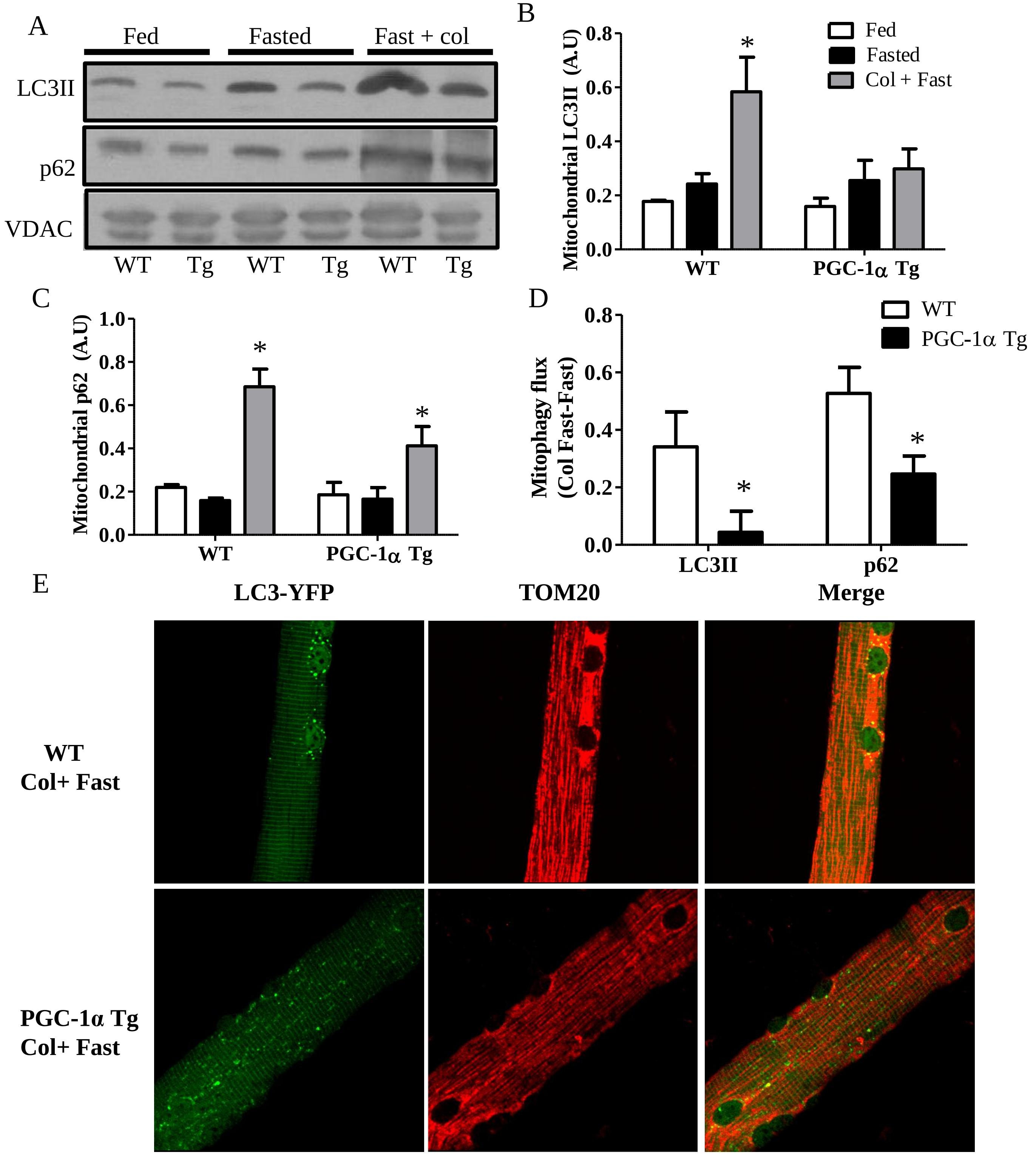

Fig. S5: Nutrient deprivation-induced Mitophagy flux is reduced in Tg animals A) Representative blots of autophagic proteins on isolated mitochondria in WT and PGC-1αTg animals that were fed or fasted, with or without colchicine (Col) treatment; B-C) quantification of protein expression and autophagy flux; B)LC3II; C)p62; D) mitophagy flux. VDAC was used as loading control. E) Single fibers isolated from the FDB muscle of animals that were fasted and treated with colchicine. Muscle was transfected with LC3-YFP (green) and immunostained for mitochondrial marker TOM20 (Red). Colocalization of LC3 with TOM20 (yellow) represents mitochondria within autophagosomes, which are more prevalent in WT animals when compared to Tg. *P<0.05 significant effect of treatment. †P<0.05 significant effect of genotype (n=3 for all groups).

Table S1. Primer sequences based on gene transcripts available in GenBank.

Gene	Forward primer (5’ → 3’)	Reverse primer (5’ → 3’)
Coxiv	CTCCAACGAATGGAAGACAG	TGACAACCTTCTTAGGGAAC
Mul1	GCCTTGCTGATGTAGTTGTCTG	CAGGCCCAGCTTATGATAGAA
Bnip3l	GGAAAGCGGCACAGAGAA	GAATGACGCCAGTGCTGAT
Park2	GTCTGCAATTTGGTTTGGAGTA	GCATCATGGGATTGTCTCTTAAA
Sqstm1	TGTGGTGGGAACTCGCTATAA	CAGCGGCTATGAGAGAAGCTAT
Maplc3b	GCTTGCAGCTCAATGCTAAC	CCTGCGAGGCATAAACCATGTA
Atg7	TTTCTGTCACGGTTCGATAATG	TGAATCCTTCTCGCTCGTACT
Lamp2	GCTGAACAACAGCCAAATTA	CTGAGCCATTAGCCAAATACAT
Catsd	TTTGCCAATGCTGTCGTACT	AGCGAGTGTGACTATGTGTGAG
Foxo3	ATGGACGACCTGCTGGATAAC	GGAGCTCTTGGCGGTATATG
Actb	TGTGACGTTGACATCCGTAA	GCTAGGAGCCAGAGCAGTAA
Gapdh	AACACTGAGCATCTCCCTCA	GTGGGTGCAGCGAACTTTAT

Table S2. Antibodies list

Antibody	Manufacturer	Product number
Goat anti-Rat Alexa Flour® 647	Life Technologies	A-21247
Goat anti-Rabbit Alexa Fluor® 488	Life Technologies	A-11034
Atg7	Sigma-Aldrich	A2856
Beclin1	Cell Signaling Technology	3738
Cathepsin D	Santa Cruz Biotechnology	Sc6486
COXIV	Abcam	Ab14744
GAPDH	Abcam	Ab8245
Histone-2B/H2B	Cell Signaling Technology	2934
Lamp-2	Abcam	Ab13524
LC3B/Maplc3b	Cell Signaling Technology	2775
NIX/Bnip3l	Abcam	Ab109414
p62/Sqstm1	Sigma-Aldrich	P0067
Parkin	Cell Signaling Technology	4211
PGC-1α/Ppargc1a	Millipore Corporation	AB3242
T-eIF2α	Cell Signaling Technoogy	9722
TFEB	MyBioSource Inc	MBS120432
Ubiquitin (Ub)	Enzo Life Sciences	ADI-SPA-203
VDAC/Porin	Abcam	14734
